# Near-Surface Studies of the Changes to the Structure and Mechanical Properties of Human Enamel under the Action of Fluoride Varnish Containing CPP–ACP Compound

**DOI:** 10.3390/biom10050765

**Published:** 2020-05-14

**Authors:** Izabela Świetlicka, Damian Kuc, Michał Świetlicki, Marta Arczewska, Siemowit Muszyński, Ewa Tomaszewska, Adam Prószyński, Krzysztof Gołacki, Jerzy Błaszczak, Krystian Cieślak, Daniel Kamiński, Maria Mielnik-Błaszczak

**Affiliations:** 1 Department of Biophysics, Faculty of Environmental Biology, University of Life Sciences in Lublin, 20-950 Lublin, Poland; siemowit.muszynski@up.lublin.pl; 2 Chair and Department of Paediatric Dentistry, Medical University of Lublin, 20-059 Lublin, Poland; damian.kuc@umlub.pl (D.K.); maria.mielnik-blaszczak@umlub.pl (M.M.-B.); 3 Department of Applied Physics, Faculty of Mechanical Engineering, Lublin University of Technology, 20-618 Lublin, Poland; m.swietlicki@pollub.pl (M.Ś.); a.proszynski@pollub.pl (A.P.); 4 Department of Animal Physiology, Faculty of Veterinary Medicine, University of Life Sciences in Lublin, 20-950 Lublin, Poland; ewarst@interia.pl; 5 Department of Mechanical Engineering and Automatics, Faculty of Production Engineering, University of Life Sciences in Lublin, 20-612 Lublin, Poland; krzysztof.golacki@up.lublin.pl; 6 Weglin-Medical, 20-716 Lublin, Poland; blaszczakjerzy@gmail.com; 7 Institute of Renewable Energy Engineering, Faculty of Environmental Engineering, Lublin University of Technology, 20-618 Lublin, Poland; k.cieslak@pollub.pl; 8 Department of Crystallography, Faculty of Chemistry, Maria Curie-Sklodowska University, 20-031 Lublin, Poland; daniel.kaminski@umcs.pl

**Keywords:** enamel surface, fluoride varnish, CPP–ACP, AFM, Raman microspectroscopy, XRD, EDS, nanoindentation

## Abstract

Changes to the features of the enamel surface submitted to induced demineralisation and subsequent remineralisation were studied. The in vitro examination was conducted on polished slices of human molar teeth, divided in four groups: the untreated control (*n* = 20), challenged by a demineralisation with orthophosphoric acid (H_3_PO_4_) (*n* = 20), and challenged by a demineralisation following remineralisation with fluoride (F) varnish containing casein phosphopeptides (CPP) and amorphous calcium phosphate (ACP) compounds (*n* = 20). The specimens’ enamel surfaces were subjected to analysis of structure, molecular arrangement, mechanical features, chemical composition, and crystalline organization of apatite crystals. Specimens treated with acid showed a significant decrease in crystallinity, calcium, and phosphorus levels as well as mechanical parameters, with an increase in enamel surface roughness and degree of carbonates when compared to the control group. Treatment with fluoride CPP–ACP varnish provided great improvements in enamel arrangement, as the destroyed hydroxyapatite structure was largely rebuilt and the resulting enamel surface was characterised by greater regularity, higher molecular and structural organisation, and a smoother surface compared to the demineralised one. In conclusion, this in vitro study showed that fluoride CPP–ACP varnish, by improving enamel hardness and initiating the deposition of a new crystal layer, can be an effective remineralising agent for the treatment of damaged enamel.

## 1. Introduction

Enamel consists on average of 95% hydroxyapatite (HA) crystals (Ca_10_(PO_4_)_6_(OH)_2_), 4% water, and 1% organic material and is characterised by the strict organic–inorganic hybrid organization. HA crystallites, along with the remaining organic material and water, are arranged into bundles. These bundles establish greater, species-dependent structures known as rods or prisms with interprism regions between them [1]. Natural HA observed in teeth can have different substitutes for Ca^2+^ (Na^+^, K^+^, and Mg^2+^) and for phosphate and hydroxyl (CO_3_^2-^, F^-^, HPO_4_^2-^, Cl^-^, and H_2_O) in comparison to those observed in the HA stoichiometric form [2,3]. The main difference between the HA model and biological apatites is the presence of significant amounts of carbonate ions in all the mineralized biological tissues [1].

In the oral environment, tooth structure undergoes alternate demineralisation and remineralisation processes, which influence structural and morphological parameters of enamel. Demineralisation is mainly caused by acids present in food, juices, or other beverages or may be a result of decreased salivary flow, gastroesophageal reflux, or eating disorders [4,5]. Remineralisation, in turn, develops as restoration of partially dissolved crystals, new crystal formation, or growth of surviving crystals [6]. Demineralization and remineralization can be considered as a dynamic processes, characterized by the flow of calcium and phosphate out of and back into tooth enamel [7]. Both remineralisation and demineralisation influence structural and morphological parameters of enamel, altering its features and changing its arrangement. Enamel is unable to regenerate, self-repair, or remodel itself as it contains no cells [8]. The natural protective mechanisms neutralizing and clearing the acids or enhancing remineralisation of enamel by providing inorganic ions [9,10] is frequently inadequate to maintain strong tissue. Then, the remineralisation process might be supported by a range of physical or chemical factors, for example, lasers [11,12], fluoride-containing treatments, substances involving casein phosphopeptides (CPP) and amorphous calcium phosphate (ACP) or a combination of them [13,14,15,16]. 

CPP–ACP is a bioactive milk product that has been found to enhance remineralization and prevents dental caries. As CPP have the ability to stabilize ACP in the metastable solution, CPP–ACP provides a pool of bio-available calcium and phosphate that can maintain the supersaturation of saliva [17]. Calcium and phosphate concentration within the subsurface lesions is kept high, which results in remineralization [18]. Fluoride ions, in turn, substitute for a hydroxyl column in the apatite structure [19]. When a tooth surface is equilibrated with oral fluids containing fluoride, the most stable phase of fluorapatite (FA) is produced, which effectively maximizes the stability of the tooth mineral in the oral environment [19]. In supersaturated solutions, where the fluoride is available together with calcium and phosphate, fluoride ions are incorporated into the apatite crystal lattice through precipitation and growth reactions. Therefore, a combination of fluoride and CPP–ACP is regarded as an optimally efficient remineralisation agent on enamel lesions when compared with a fluoride treatment alone [20]. New, rebuilt crystalline structures, composed of fluoridated hydroxyapatite and fluorapatite, are characterised by a higher resistance to acid attack than the original ones [20].

The aim of this study was to examine in vitro the effect of a fluoride varnish containing CPP–ACP on the remineralization of artificially demineralized human enamel. As the action of chemical substances supporting the remineralisation process is mainly visible on the tooth surface, therefore, in the presented work, we wanted to concentrate particularly on the enamel surface. To recognise the enamel’s surface features in a maximally wide range, we tested its morphology, chemical composition, mechanical properties, crystal and molecular arrangement after demineralisation, and subsequent remineralisation with CPP–ACP fluoride varnish measurements involving atomic force microscopy (AFM), scanning electron microscopy (SEM), energy-dispersive X-ray spectroscopy (EDS), Raman microspectroscopy, X-ray diffraction (XRD) technique, and nanoindentation. 

## 2. Materials and Methods 

Examinations were conducted on slices of human third molar teeth that belonged to the control group, were demineralised with an orthophosphoric acid (H_3_PO_4_), or were demineralized and then underwent remineralisation under the action of fluoride varnish (MI Varnish, Tokyo, Japan) containing casein phosphopeptides and amorphous calcium phosphate. 

**Figure 1:**
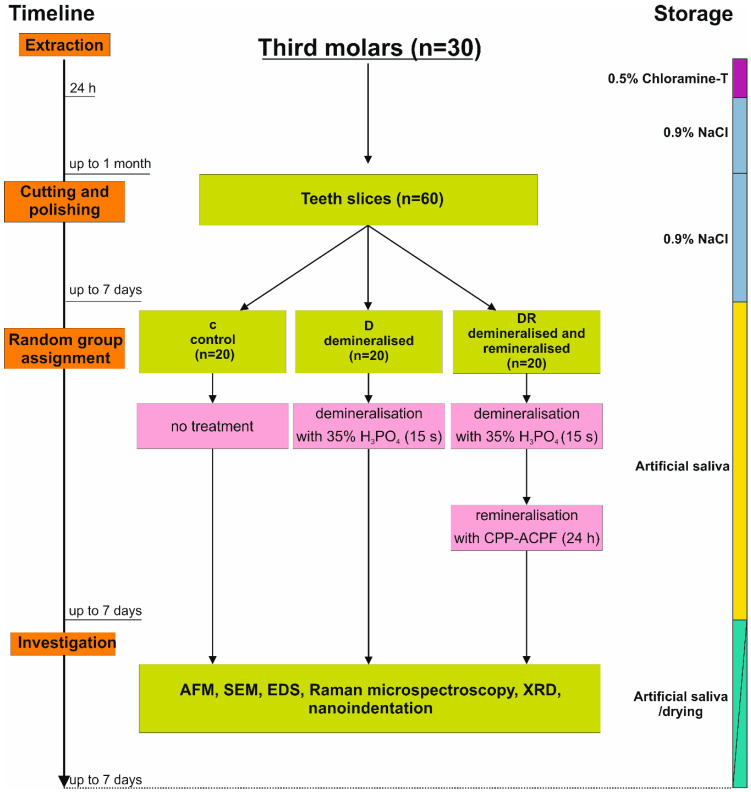
Experimental design. c—control, D—demineralised group, DR—demineralised and then remineralised group.

Teeth were extracted for orthodontic reasons and were caries-free. All subjects gave their informed consent for inclusion before they participated in the study. The study was conducted in accordance with the Declaration of Helsinki, and the experiment was approved by The Local Ethics Committee at the Medical University of Lublin (reference number KE-0254/257/2017) on 26 October 2017. After preparation, the enamel surfaces were subjected to multidimensional analysis. The detailed description of the experimental procedures is provided below and can be tracked in the scheme (Figure 1).

### 2.1. Sample Preparation

**Figure 2:**
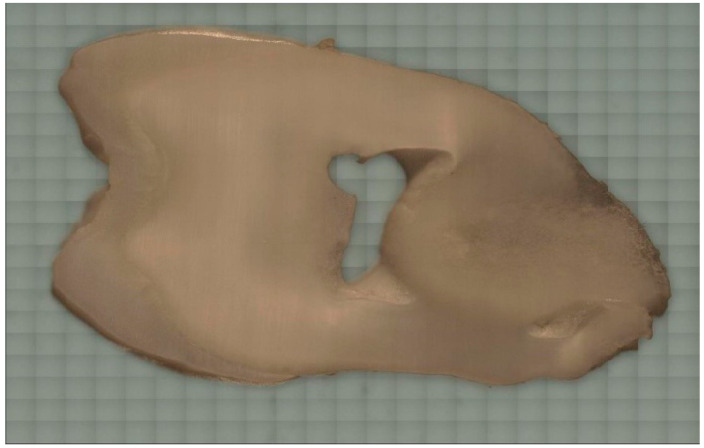
Example slice of human molar.

Extracted teeth were disinfected in aqueous 0.5 % Chloramine-T solution (Poch S.A., Gliwice, Poland) dissolved in deionized water at 4 °C for 24 h, washed with deionized water, dried in a desiccator [21], and placed in a 0.9 NaCl solution at 4 °C for no longer than a month. Prepared molars (*n* = 30) were cut longitudinally into slices (2–4 from each molar) with an average thickness of 1.5 (± 0.025) mm using a low-speed diamond saw (Figure 2). To obtain a flat, scratch-free surface, teeth slices were polished with 1500, 2000, 2500, 3000, and 6000 grit waterproof silicon carbide paper, followed by 3, 2, and 1 mm aluminium oxide, and then cleaned and washed for 20 s in distilled water. Samples with cracks or other defects were removed from the experiment. The cut fragments were stored in a 0.9 % NaCl solution at 4 °C until treatment (demineralisation and remineralisation), for no longer than 7 days. Teeth slices were divided into three sets (*n* = 20 for each group) according to their pre-treatments before testing: the first group of slices belonged to the control (c) and for that group no treatment was applied, those in the second group were demineralised (D), while those in the third were demineralised and next remineralised (DR). Demineralisation was done by the immersion of the molar slices for 15 s in 35% orthophosphoric acid (H_3_PO_4_), which is known to cause dissolution of enamel minerals and is widely applied in studies concerning carries simulation [15,22]. Next, slices were rinsed by deionized water (Millipore, Bedford, MA, USA) for 20 s [23]. Teeth from the other group, after demineralisation, were covered with varnish according to the manufacturers’ instructions and left for 24h in a moist environment (artificial saliva, pH 7.0 [24]). After this time, the varnish was removed from the surface and the samples were cleaned with cotton swabs soaked in acetone and rinsed with deionised water [13,25]. 

Obtained teeth slices from the experimental groups were stored at 4 °C in artificial saliva (changed daily) and were subjected to analysis within no longer than 7 days. 

### 2.2. AFM Measurements and Roughness Calculation

Examination was undertaken on *n* = 30 teeth slices (*n* = 10 for each group). Tooth fragments, previously dried in a desiccator under vacuum conditions for 24 h, were mounted on the sapphire plates by an adhesive tape. Enamel surfaces were initially examined under an optical microscope to determine three regions intended for AFM measurements. The measurements were carried out under atmospheric conditions in a semi-contact mode, at room temperature, and with relative humidity of 25% by NTEGRA Prima (NT–MDT, Moscow, Russia). Silicon cantilevers 125 μm long, 4.0 μm thick, with a tip radius of 10 nm, and the average resonant frequency of 310 kHz were used. For each slice, three areas in size 30 μm × 30 μm were investigated with a slow scan rate of 1 Hz and with a resolution of 512 × 512 pixels per image. The enamel surface was pictured in the height, magnitude, and phase domains. The parameters describing surface roughness (average roughness S_a_, root mean square roughness S_q_, the vertical distance between the maximum height and the maximum depth S_z_, and the average height calculated over the five highest peaks p and five deepest valleys v S_10_) were determined for a height function Z_ij_(x,y) defined over a certain XY plane, according to Equations (1) to (4):

(1) $$S_{a}=\frac{1}{N_{x}N_{y}}\sum_{j=1}^{N_{y}}\sum_{i=1}^{N_{x}}\left|Z_{ij}\right|,$$

(2) $$S_{q}=\sqrt{\frac{1}{N_{x}N_{y}}\sum_{j=1}^{N_{y}}\sum_{i=1}^{N_{x}}Z_{ij}^{2}}\;,$$

(3) $$S_{z}=S_{p}+S_{v},$$

(4) $$S_{10}=\frac{\sum_{i=1}^{5}\left|Z_{pi}\right|+\sum_{i=1}^{5}\left|Z_{vi}\right|}{5},$$

where N_x_ and N_y_ are the sampling rates along the X and Y axes, respectively, S_p_ is the maximum height of the surface defined with respect to the mean surface, and S_v_ is defined as the maximum depth of the surface defined with respect to the mean surface [26,27]. 

### 2.3. SEM Imaging and EDS Measurements

To perform the morphological analysis of enamel, a scanning electron microscope (SU3500, Hitachi Ltd., Tokyo, Japan) working at 20 kV in high vacuum conditions was used. Prior to introducing the samples to the SEM chamber (*n* = 3 from the control, *n* = 3 demineralised, and *n* = 3 demineralised then remineralised), slices were dried in a desiccator for 24 h, and then coated with an ultrathin gold (Au) film with ion sputtering equipment (Sputter Coater 108auto, Cressington Sci. Instr., Watford, UK). The enamel surface was imaged with 3000× and 8000× magnification.

In order to establish the element composition of the teeth slices, during SEM, imaging energy-dispersive spectrometry (EDS) was performed. An UltraDry high-performance X-ray energy dispersive Silicon Drift detector (Thermo Scientific Noran System 7, Thermo Electron Scientific Instruments LLC, Madison, WI, USA) was used. The detector was operated in a high vacuum at 20 kV with a working distance 10 mm. X-ray spectra were acquired and analysed in terms of percentage composition of elements included in the teeth, especially Ca, P, and F. 

### 2.4. Raman Spectra Collection and Data Analysis

The Raman spectra were collected on a NTEGRA Spectra confocal spectrometer (NT–MDT, Moscow, Russia) under excitation with a diode laser operating at a wavelength of 532 nm. The incident laser power applied to the sample was 16 mW and a 600 lines/mm grating with a blaze wavelength of 600 nm was applied. A ×100/0.7 objective was used to focus the laser light on the sample while a charge-coupled device (CCD) camera (1650 × 200 pixel matrix), operating at 230 K, was applied to detect the scattered light. Raman maps were recorded with a sampling frequency of 0.25 μm in both *x* and *y* directions. For each tooth (*n* = 3 for each group), two maps of size 5 μm × 5 μm each were acquired. All the Raman spectra were collected within a spectral range of 100–2000 cm^−1^. The spectral resolution amounted to the average of 2.44 cm^−1^. Individual Raman spectra were analysed using the Grams/AI 8.0 (Thermo Scientific, Waltham, MA, USA). Due to the high dependence of Raman spectra from the HA crystallites orientation in the *c*-axis [28,29,30,31], further analysis spectra from the rod regions only were selected [32]. All Raman spectra were corrected from the fluorescence input using a polynomial baseline function, smoothed by a 5-point Savitzky–Golay filter, and normalized using the 960 cm^−1^ (the ν_1_ mode) band characterized by its relative intensity. The band positions were performed according to Gaussian fitting functions after subtracting a polynomial baseline. 

### 2.5. Single Crystal X-Ray Diffraction

The samples (*n* = 9, 3 for each group), were dried for 24 h before testing in vacuum conditions. Enamel surfaces were measured in a θ–2θ geometry (Rigaku XtaLAB, Tokyo, Japan). The diffractometer was equipped with a MicroMax–007 HF rotated anode X-ray source and a Pilatus 300 K area detector, working over a range of 6-110 deg with a resolution of 0.078 deg and 10 min counting time per frame. The diameter of the X-ray beam was 0.1 mm. The sample was rotated around the direction perpendicular to the θ–2θ plane (around the *z* direction) for signal averaging from 0.1 mm sample slices. The measured frames were processed in CrysAlisPRO software (Rigaku, Tokyo, Japan). The Scherrer equation (Equation (5)) [33] was used to calculate the mean size of the nanocrystallites:

(5) $$D=\frac{K\lambda }{\beta cos\theta },$$

where D is the mean size of the ordered crystalline domains, K is a constant related to the crystallite shape (0.9), β is the full width of the peak at half of the maximum intensity (FWHM), counting the apparatus broadening of 0.08 deg (limited by the detector resolution at 40 mm from the sample), λ is the wavelength of X-ray radiation (1.5407 Å), and θ is the peak position. The sizes of the crystallites in the c crystallographic direction (z-axis) were calculated from the Miller index (002) [2,34]. Lattice spacing (interplanar spacing) d_hkl_ was determined from the Bragg’s law for the Miller indices (300) and (002) according to Equations (6) and (7):

(6) $$\lambda =2d_{hkl}sin\theta_{hkl}$$

(7) $$\frac{1}{d_{hkl}^{2}}=\frac{4}{3}\left(\frac{h^{2}+hk+k^{2}}{a^{2}}\right)+\frac{l^{2}}{c^{2}}$$

where h, k, and l are the Miller indices that are the reciprocal intercepts of the plane on the unit cell axes [2]. Lattice parameters a and c for the hexagonal unit cell were calculated to assess the type of the apatite (HA or FA) present in the enamel structure after remineralization. Furthermore, the crystallinity index (CI), which measures the percentage of crystalline material and is correlated to the degree of order within the crystals [35], was determined using Equation. (8):

(8) $$CI={\left(\frac{K_{A}}{FWHM_{002}}\right)}^{3},$$

where K_A_ is a constant set to 0.24 and FWHM_002_ is the full width of the peak at half intensity of the (002) reflection peak [35]. Crystallographic planes and Bragg peaks were calculated using Mercury CSD 3.10.1 software (CCDC, Cambridge, UK) from the hydroxyapatite references (No. 2300273, Crystallography Open Database, and No. 96-901-0053, High Score Plus package software). The peak position and FWHM were determined from the Gaussian function fits to every peak with OriginPro 2016 software (OriginLab Co., Northampton, MA, USA) application. 

### 2.6. Nanoindentation Measurements

Slices of molar teeth were dried in vacuum conditions prior to examination in order to avoid the adhesion effect [36]. The nanoindentation tests were carried out using the Ultra Nano Hardness Tester (UNHT CSM Instruments, Needham, MA, USA) with a diamond Berkovich indenter with a face angle of 65.3 deg (±0.3). The indentations were taken at room temperature using the linear loading mode with a max depth of 400 nm, 30 mN/min loading rate, and pauses of 10 s. For a given penetration depth value, ten indentations were made for each sample (*n* = 15, 5 for each group), and the average values of indentation hardness (HIT) and indentation elasticity (EIT) were calculated. 

### 2.7. Data Analysis

Statistical analysis was performed using Statistica13.1 (TIBCO Software Inc. Palo Alto, CA, USA) and OriginPro 2016 (OriginLab Co., Northampton, MA, USA) applications. After removal of outliers, the resulting dataset was checked for normality distribution by the Shapiro–Wilk test, while the homogeneity of the variance was checked using the Levene test. One-way ANOVA was applied to assess the differences among the examined groups (control (c), demineralised (D), and demineralised then remineralised (DR)) in terms of the parameters characterising the structure and organisation of the enamel surface. The significance level *p* was set on 0.05. After analysis, further post-hoc tests (Tukey) were carried out to ascertain the nature of the differences among groups. 

## 3. Results

### 3.1. Morphology and Chemical Composition

**Figure 3:**
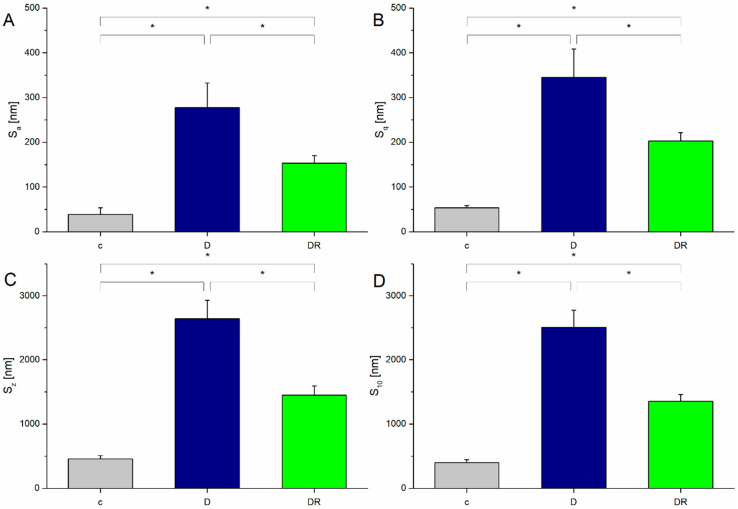
Roughness parameters of the enamel surface for the control (c), demineralised (D), and demineralised then remineralised (DR) enamel: (**A**) the average roughness S_a_, (**B**) the root mean square roughness S_q_, (**C**) the vertical distance between the maximum height and the maximum depth S_z_, (**D**) the average height calculated over the five highest peaks and five deepest valleys S_10_. Roughness parameters were determined over a surface of 30 µm × 30 µm. Statistically significant (at *p* < 0.05) differences between groups are marked with *.

The process of enamel surface treatment with an orthophosphoric acid caused a significant increase in surface roughness (Figure 3), indicating that the enamel’s surface morphology underwent considerable changes. The average values of the roughness parameters in the demineralised group were about 6.5 times higher those of the control. 

The averaged values of the roughness measurements for each examined tooth slice are in Figures S1–S4.

**Figure 4:**
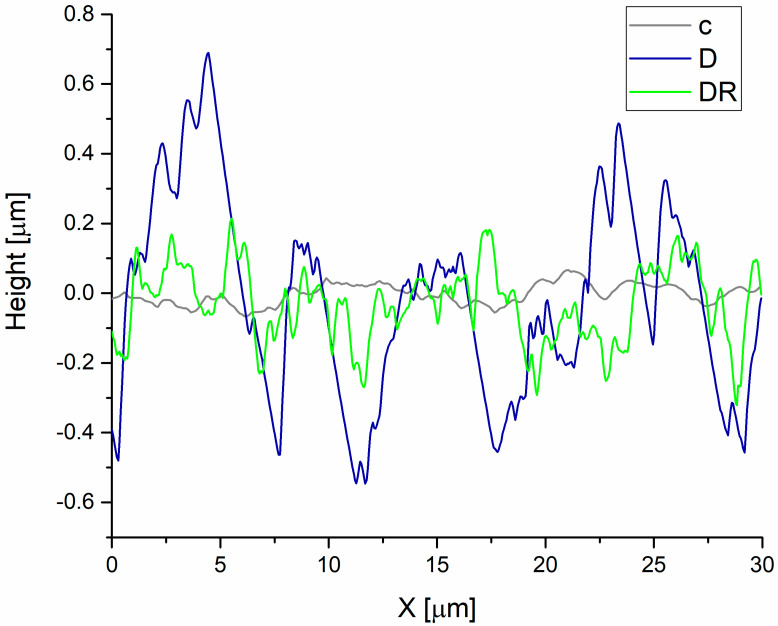
The average height profile for the control (c), demineralised (D), and remineralised then demineralised (DR) enamel over the scanning distance of 30 µm.

**Figure 5:**
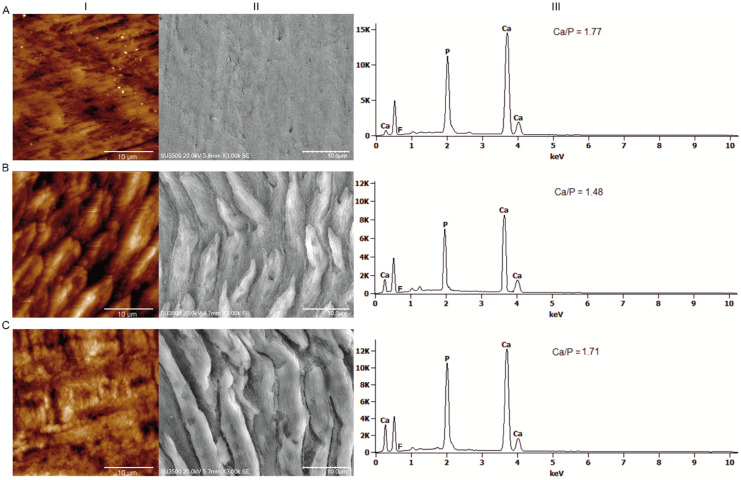
AFM (**I**) and SEM (**II**) scans of the enamel surface from the control (**A**), demineralised (**B**), and demineralised then remineralised (**C**) groups with corresponding EDS spectra (**III**) acquired from the enamel surfaces showing differences in the Ca/P ratio for the examined groups. The Ca/P ratios are in Figure S5. a, b—groups that do not differ at *p* < 0.05.

When the height profile over the scanning distance (30 µm) was considered (Figure 4), it was clear that the acid-treated enamel surface became more irregular and asymmetrical. The maximum difference in the height for the control group was equal to 0.13 µm, while for the demineralised group it was 1.24 µm. Additionally, a registered demineralisation pattern (Figure 5, IB and IIB) revealed that the interprism regions and partially prism cores were damaged, which led to the occurrence of gaps and cracks on the enamel surface.

Corresponding EDS spectra acquired from the enamel surfaces registered a significantly lower Ca/P ratio in the D group compared to the control (Figure 5, IIIA, and IIIB). Varnish application resulted in a significant resmoothing of the enamel surface. The values of the roughness parameters for the DR group were about 1.8 times lower compared to those of the demineralised one (Figure 3). The enamel surface became more regular and organised, which can be observed on the height profiles (Figure 4) and both the AFM and SEM scans (Figure 5, IC and IIC). However, the roughness was still notably higher compared to the control. A significant difference in the roughness parameters was caused by an appearance of amorphous material deposits on the enamel surface, which are clearly visible in Figure 5, IIC. The maximum difference in the height over the scanning distance for the DR group was reduced to 0.54µm. Additionally, EDS spectra showed an increase in the Ca/P ratio compared to the demineralised group (Figure 5, IIIC).

### 3.2. Structural Characterisation

#### 3.2.1. Raman Spectroscopy Results

**Figure 6:**
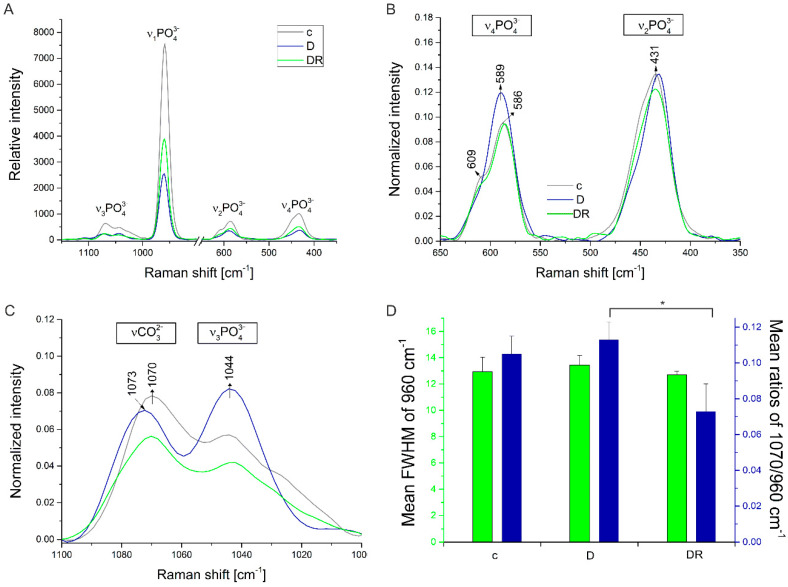
Averaged (*n* = 10 to each group) Raman spectra of dental enamel (**A**) from the control group (black line), after acid etching (blue line) and after the casein phosphopeptides and amorphous calcium phosphate (CPP–ACP) treatment (green line), (**B**) for ν_2_ and ν_4_ modes, (**C**) ν_3_ mode, of B-type carbonate and (**D**) corresponding full-widths at half maximum of the 960 cm^-1^ peak and the ratio of the integrated area under the bands of 1070 and 960 cm^-1^. Statistically significant (at *p* < 0.05) differences between groups are marked with *. c—control, D—demineralised group, DR—demineralised—then remineralised group.

In all groups, the Raman spectra of tooth enamel were dominated by phosphate bands of which there are four principal PO_4_^3−^ internal vibrational modes for apatite labelled ν_1_ to ν_4_ (Figure 6A). The most intensive ν_1_ PO_4_^3−^ transition (~960 cm^−1^) arises from the symmetric P–O bond stretches in the mineral apatite component of enamel. The ν_2_ region of the spectrum, mainly a band located at ~430 cm^−1^ is assigned to the symmetric bending mode of PO_4_^3−^. The other very weak bands in the spectrum are the ν_3_ of phosphate (~1040 cm^−1^) and the ν_1_ of B-type carbonate (~1070 cm^−1^), where PO_4_^3−^ is substituted by CO_3_^2−^. Finally, the bands located at ~580 and 608 cm^−1^, which belong to the ν_4_ vibrational modes, arise from the triply degenerated asymmetric bending vibrations [37].

Symmetric bending vibrations of PO_4_^3−^ in the region of the ν_2_ mode appeared as a band at ~430 cm^−1^ with a shoulder at 445 cm^−1^ for the control group (Figure 6B). After acid etching, a much sharper peak at 430 cm^−1^ was observed (Figure 6B). Comparing the spectra in the range of 650–350 cm^−1^, it was found that the intensity of the phosphate ν_4_ band slightly increased and revealed the spectral shift towards higher frequencies after acid treatment of enamel samples (Figure 6B). Interestingly, Raman spectra of enamel subjected to the varnish appeared very similar to the demineralised ones.

Loss of structure in the demineralised enamel samples was indicated by the low intensity of the ν_1_ CO_3_^2−^ band (Figure 6C). In the ν_3_ PO_4_^3−^ region, the band centred near 1044 cm^−1^ was more intense than the band centred near 1070 cm^−1^, which suggested the superposition of a ν_1_ CO_3_^2−^ band (Figure 6C). The mineral content, which is demonstrated by the intensity of the peak at 960 cm^−1^, decreased after demineralization (Figure 6A). Although there was no statistically significant difference between the investigated samples in the full-width at half maximum of the 960 cm^-1^ peak (Figure 6D), the intensity of the ν_1_ PO_4_^3−^ band was almost ten times less for demineralized enamel than those seen for untreated enamel.

The ratio of the integrated area under bands 1070 and 960 cm^−1^ (1070/960 cm^-1^) was used to analyse the carbonate content (Figure 6D). It revealed an increase in this ratio in the case of the demineralized sample when compared to that of the control group. The carbonate content evaluation exhibited a significant reduction in the experimental group subjected to the remineralization process with CPP–ACP treatment.

#### 3.2.2. XRD Pattern Analysis

**Figure 7:**
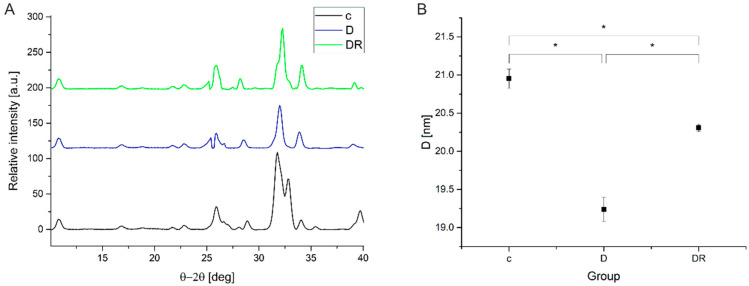
X-ray diffraction pattern of enamel hydroxyapatite (HA) crystals (**A**) and mean crystallite size of HA crystallites in the z crystallographic direction (**B**) with corresponding standard deviations. Significantly different groups (at *p* < 0.05) are marked with *. c—control, D—demineralised group, DR—demineralised then remineralised group.

Reflection peak at 2θ = 25.886 deg (002) from XRD patterns (Figure 7A), was used to determine HA crystallites length in the z-axis (Figure 7B) and crystallinity index (Table 1) for all the examined groups. Reflection peaks at 2θ = 32.912 deg (300) and 2θ = 25.886 deg (002) were used to calculate the hexagonal lattice parameters for the control (c), demineralised (D), and demineralised then remineralised (DR) enamel (Table 1). Acid treatment caused perceptible shortening of crystallites in a *z*-crystallographic direction along with a significant (*p* < 0.05) decrease in the crystallinity value (Table 1). Varnish application (the DR group) lead to HA crystallites lengthening, however, they were still notably shorter than those of the control group. For the CI index (Table 1), the significant increase after varnish treatment was registered.

No significant differences were observed in the features of the lattice parameters among the groups studied. Only for the demineralised enamel significant (*p* = 0.0021) shortening of the c lattice parameter was registered (Table 1).

#### 3.2.3. Mechanical Features

**Figure 8:**
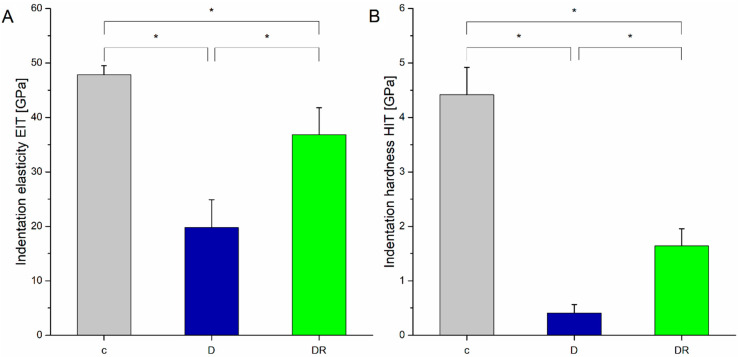
Indentation elasticity (**A**) and indentation hardness (**B**) for the control (c), demineralised (D), and demineralised then remineralised (DR) enamel surfaces. Statistically significant differences (at *p* < 0.05) are marked with *. Raw data are in Figures S6 and S7.

Demineralisation caused a significant decrease (*p* < 0.001) both in indentation elasticity (EIT) and indentation hardness (HIT), as presented in Figure 8A and 8B, respectively. Although still lower in comparison to the control, enamel surface was enhanced after varnish remineralisation, showing the increase in both investigated parameters. A 288% growth in HIT and a 67% growth in EIT were registered for the DR group in relation to the demineralised one. The enamel surface had become more rigid and more resistant against deformation, as the measurements revealed.

## 4. Discussion

The presented study showed that, after 24 h, fluoride varnish with CPP–ACP induced the partial regeneration of previously damaged enamel tissue, activating the formation of a new crystal layer. It was observed that the destroyed hydroxyapatite structure was largely rebuilt and the honeycomb-like structure was partially restored. The resulting surface was characterised by the greatest regularity, higher molecular and structural organisation and smoother surface when compared to that of the demineralised sample. Significant alterations in the morphology, mechanical properties, and chemical and molecular structures indicate that the remineralisation was the result of a range of processes observed on the surface layer of the enamel tissue.

A crystal is made up of several unit cells that are stacked in multiples along all possible axes and determine the crystal shape [38]. As observed in the presented research, acid-induced dissolution of minerals remodels the HA crystallite form and lowers the crystallinity index [10]. Increased disorder of the crystal structure and reduction of the crystallinity, as demonstrated by the broadening of the diffraction peaks, are not only the measure of the degree of mineralisation but are also connected with higher numbers of planar carbonate ions substituting for the tetrahedral phosphate ions in the apatite structure [39]. This is biologically important because an increase in carbonate content means an increase of the solubility of the dental apatite [40] and consequently formation of calcium deficient apatite. In light of the reported data regarding the correlation of carbonate content with enamel caries appearance, the high level of carbonate is thought to have a destabilizing effect [41] and may lead to creation of unstable apatite phases [42]. Therefore, lowering of the enamel crystallinity of our samples could be related to the observed increase in the CO_3_^2−^/PO_4_^3−^ band ratio.

Remineralization is the process of transferring anions and cations to nucleation sites, where the lattices, leading to mineral structures, are generated [10]. The lengthening of HA crystallites after varnish action (Figure 5 IC and IIC, Figure 7) indicates, that the remineralisation process resulted in the reconstruction of damaged HA crystallites and growth of new ones [6]. The above observations are also proven by the changes in FWHM of the (002) reflection peak and the degree of order of the crystalline matrix measured by the crystallinity index [43]; with higher CI indices, more ordered and more mineralised tissue might be observed. Additionally, the carbonate content evaluation, with regard to the integrated area under bands (1070/960 cm^−1^) calculated from Raman spectra, exhibited a significant reduction in concentration in CO_3_^2−^ in experimental group subjected to a remineralization process with varnish treatment. 

Alterations in the crystalline structure directly translate into changes in the surface texture [44]. With respect to the calculated roughness parameters (Figure 3) and corresponding AFM and SEM scans (Figure 5I and II), varnish action led to the regeneration of a more uniform and compact surface layer. The characteristic enamel pattern had been largely rebuilt, resulting in a more homogenous surface. Though the enamel surface observed for the remineralised group was smoother compared to that of the eroded one, registered roughness was still higher compared to that of the control group. Detected fluctuations of roughness parameters for remineralised enamel are probably connected with the noticed amorphous material deposits [24,44,45]. 

It has been reported that fluoride substances containing CPP–ACP are characterised by the highest fluoride and calcium ion release compared with other calcium phosphate substances and fluoride varnishes alone [46,47]. In supersaturated solutions, where the fluoride is available together with calcium and phosphate, fluoride ions are incorporated into the apatite crystal lattice [47], and form FA. They replace the hydroxyl ions located at the centre of the calcium triangles. While the hydroxyl ion is too large to fit into this place, the oxygen is displaced from the plane of the calcium triangles and the F ions are surrounded by calcium ions. This results in a reduction in the volume of the unit cell [47] (HA: a = b = 9432 Å and c = 6881 Å; FA: a = b = 9367 Å and c = 6884 Å [48]) and contributes to a more compact lattice and stronger electrostatic bonds between calcium and F ions than between calcium and OH ions [49]. FA is more stable than HA and more resistant against acids [48], which can be translated into changes in the mechanical features. The observed increase in the indentation hardness and indentation elasticity after varnish action may suggest the OH^-^ substitution by F^-^. Nevertheless, the values of lattice parameters and spectroscopy measurements contradict the occurrence of that process. Unit cell dimensions as well as low fluoride levels suggest that OH groups in the HA structure have not been replaced by fluoride ions, or the range of the process was not extensive. A growth in both the hardness and the elasticity revealed by nanoindentation measurements may therefore be associated with mentioned reconstruction of the HA structure and the growth of the new, strong crystals. 

It should be noted, that mechanical parameters of tooth enamel are strongly related to their mineral content [5,50,51]. Loss of the minerals induced by acid application is known to cause a reduction in the hardness and elasticity of the enamel surface [44,52,53]; this was also noted in the present study. It was found that artificial demineralization, recognised as a reduction in crystallographic texture (preferred orientation), was coupled with the loss of mineral mass [54]. As mentioned, the main building blocks of enamel are calcium and phosphate ions, thus, the determination of their content allowed us to notice a significant decrease in the Ca/P ratio after acid treatment. The reduction of the Ca/P ratio was primarily due to a decrease in the amount of calcium ions in the hydroxyapatite structure, these ions are notably vulnerable to orthophosphoric acid during the first few minutes of its action [55]. The use of varnish caused a significant rise in the mineral content and return of the Ca/P ratio to the initial value. The observed effect is probably directly connected to casein features, as its micelles serve as carriers of calcium phosphate, providing the tissue with a bioavailable source of calcium and phosphate ions [56], thereby increasing the extent of tissue calcification [57]. What is more, casein phosphopeptide can deliver amorphous calcium phosphate and help the ACP to bind with dental enamel [58], supplying it with the necessary elements. A similar pattern concerning Ca and P content changes, as a consequence of enamel demineralisation and then remineralisation, was observed by Zhang et al. [6], where the morphological, chemical, and crystallographic characters of the remineralised surface were investigated. Our measurements showed that for the non-treated enamel, the Ca peak observed in the EDS spectra had a higher intensity than the P peak, while after demineralisation, both peak maximums were close to each other. In contrast, remineralisation brought the contents of elements almost to their initial state, hence, CPP–ACPF was found to re-harden the acid-treated enamel, however, it was not possible to recover it to its previous form. 

According to most of the literature, the use of preparations containing CPP, ACP, and F causes an increase in mechanical parameters of enamel [44,52,53], in contrast to [59] in which a similar effect was not observed. This is probably related to the types of mechanical tests used and the range of erosion. Shorter acid exposure times cause less erosion depth in enamel tissue. Microhardness or bending tests are not able to detect differences in mechanical parameters if the depth of erosion is in the nanometre scale [60].

While our findings provide evidence of how fluoride substances containing natural compounds in the form of CPP–ACP act on the enamel surface, the presented work had some limitations that require comment. Primarily, the presented research involved in vitro studies and cannot be directly translated into a real conditions. Despite the that fact that the prevailing environment of the oral cavity was mimicked to a large extent, the range of the introduced effects may differ from those shown. Another potential weakness of our study is related to sample preparation. The enamel surfaces examined in this in vitro experiment belong to the inner part of enamel tissue, which, contrary to the prismless and highly mineralised surface of permanent teeth, is characterised by a prismatic structure and lower mineralization level [61,62]. It might be assumed that the dynamics of acid and varnish action may differ [63]. The processes of cutting and polishing were, nonetheless, indispensable as they made the enamel samples more uniform.

## 5. Conclusions

CPP–ACP fluoride varnish acted as an instigator of the remineralisation process, initiating the formation of a new crystal layer with parameters similar to those of the initial enamel. Surface analysis conducted revealed significant alterations in the morphology, mechanical properties, and chemical and molecular structures of the studied enamel tissue as a result of the processes that occurred on the surface layer.

## Figures and Tables

**Table 1:** Mean values of enamel crystallinity index (CI) with corresponding standard deviations (SD).

Group	CI	Lattice Parameter a = b (nm)	Lattice Parameter c (nm)
c	0.238 ^a^ (±0.004)	0.94559 ^a^ (±0.00062)	0.68803 ^a^ (±0.00024)
D	0.185 ^b^ (±0.005)	0.94525 ^a^ (±0.00081)	0.67816 ^b^ (±0.00086)
DR	0.221 ^c^ (±0.002)	0.94512 ^a^ (±0.00031)	0.68821 ^a^ (±0.00023)

^a, b, c^—groups that do not differ at *p* < 0.05; c—control, D—demineralised group, DR—demineralised then remineralised group.

## Supplementary Materials

The following are available online at https://www.mdpi.com/2218-273X/10/5/765/s1, Figure S1: An averaged values of the average roughness (Sa) for the control (c) demineralised (D) and demineralised-remineralised (DR) enamel with corresponding standard deviations (whiskers) and mean values (black square), Figure S2: An averaged values of the root mean squared roughness (S_q_) for the control (c) demineralised (D) and demineralised-remineralised (DR) enamel with corresponding standard deviations (whiskers) and mean values (black square), Figure S3: An averaged values of the vertical distance between the maximum height and the maximum depth (S_z_) for the control (c) demineralised (D) and demineralised-remineralised (DR) enamel with corresponding standard deviations (whiskers) and mean values (black square), Figure S4: An averaged values of the average height calculated over five highest peaks and five deepest valleys (S_10_) for the control (c) demineralised (D) and demineralised-remineralised (DR) enamel with corresponding standard deviations (whiskers) and mean values (black square), Figure S5: Ca/P ratios determined for the analysed samples from the control (c), demineralised (D) and demineralised-remineralised (DR) groups with corresponding standard deviations (whiskers) and mean values (black square), Figure S6: Indentation elasticity (EIT) values determined for the analysed samples from the control (c), demineralised (D) and demineralised-remineralised (DR) groups with corresponding standard deviations (whiskers) and mean values (black square), Figure S7: Indentation hardness (HIT) values determined for the analysed samples from the control (c), demineralised (D) and demineralised-remineralised (DR) groups with corresponding standard deviations (whiskers) and mean values (black square).
