# Supplementary material for: Near-Surface Studies of the Changes to the Structure and Mechanical Properties of Human Enamel under the Action of Fluoride Varnish Containing CPP–ACP Compound
Source: Biomolecules. 2020 May 14;10(5):765. doi: 10.3390/biom10050765 (PMC7277937; doi:10.3390/biom10050765)
Supplement: Supplementary file 1 [file biomolecules-10-00765-s001.pdf]

Supplementary material

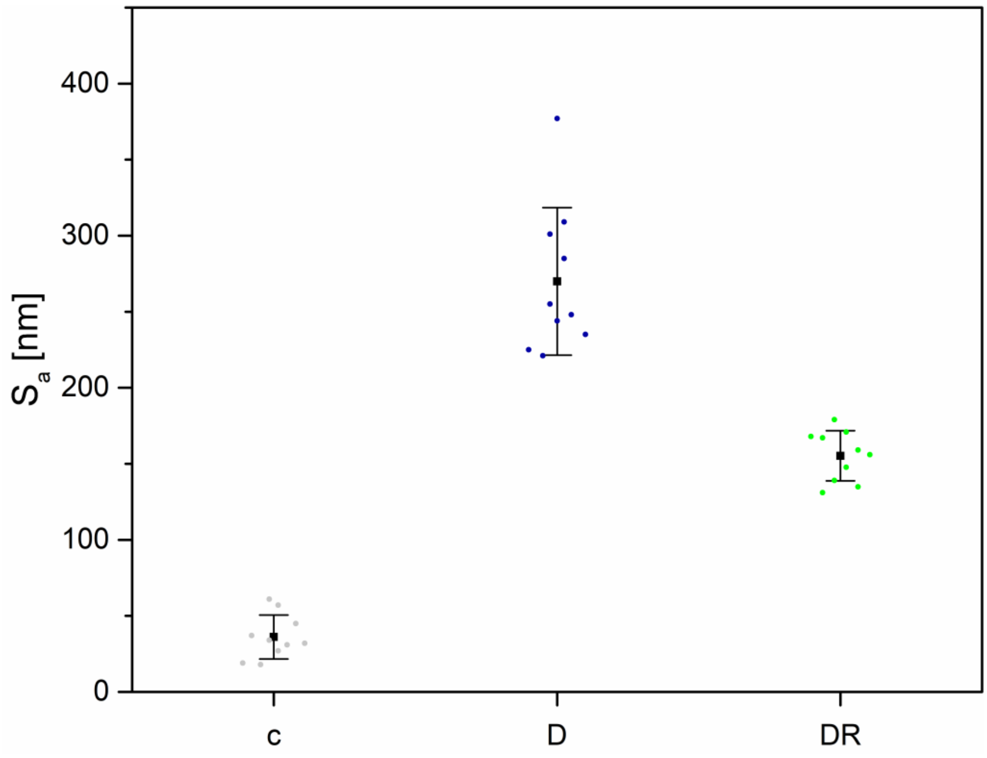

Figure S1: An averaged values of the average roughness (S_a) for the control (c) demineralised (D) and demineralised-remineralised (DR) enamel with corresponding standard deviations (whiskers) and mean values (black square).

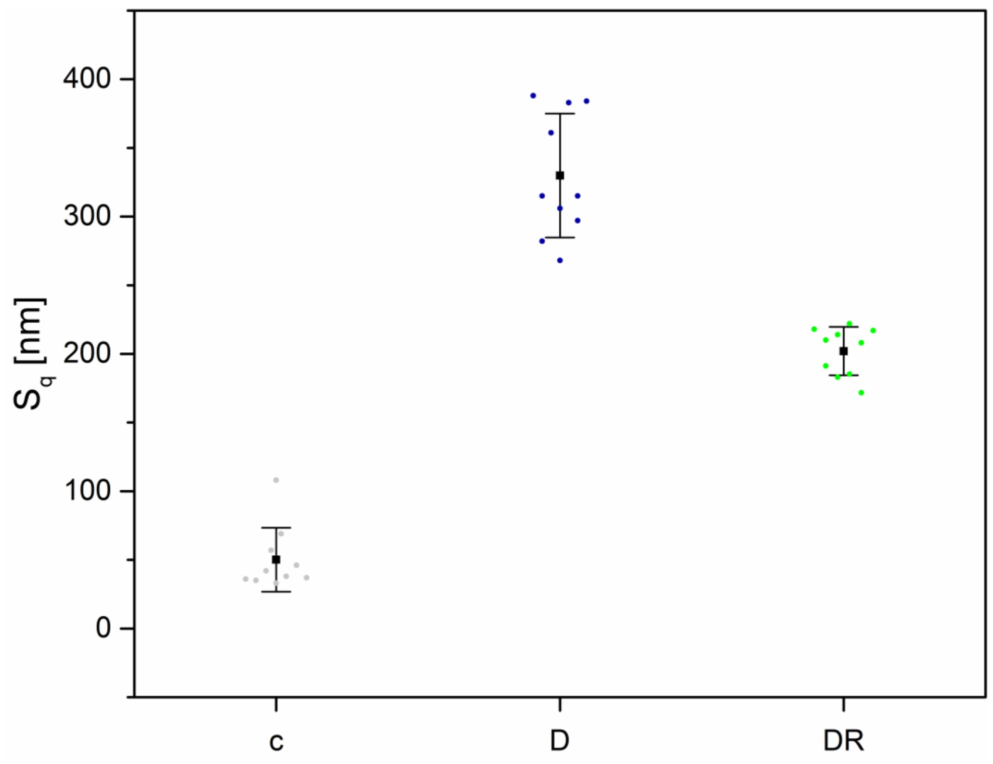

Figure S2: An averaged values of the root mean squared roughness (S_q) for the control (c) demineralised (D) and demineralised-remineralised (DR) enamel with corresponding standard deviations (whiskers) and mean values (black square).

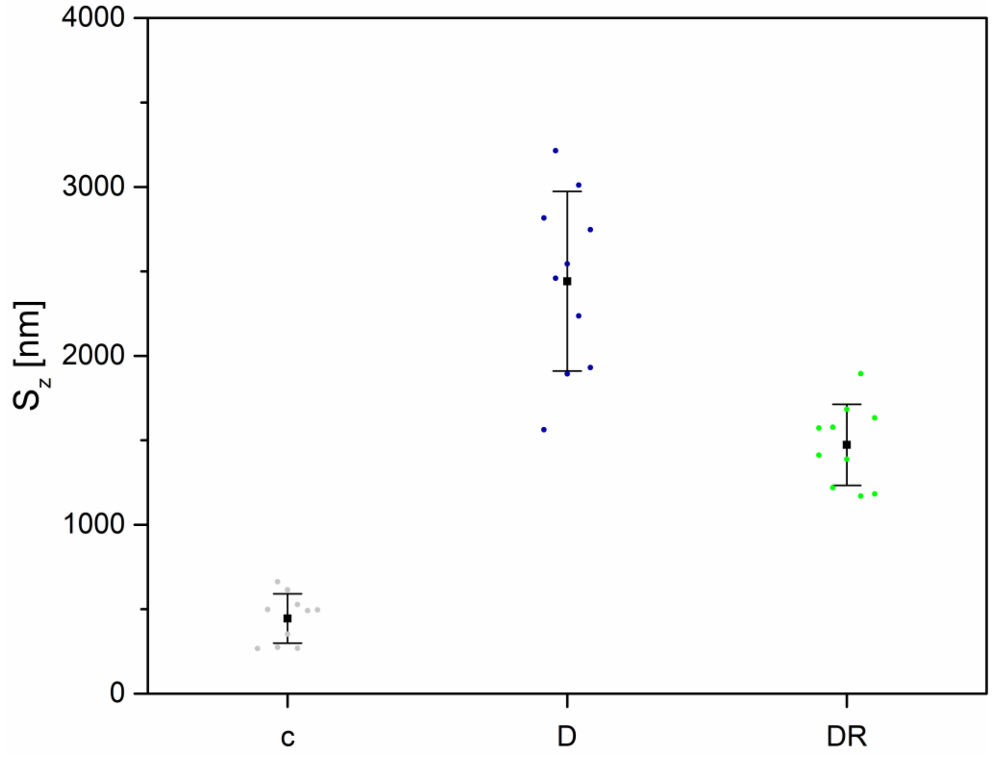

Figure S3. An averaged values of the vertical distance between the maximum height and the maximum depth (S_z) for the control (c) demineralised (D) and demineralised-remineralised (DR) enamel with corresponding standard deviations (whiskers) and mean values (black square).

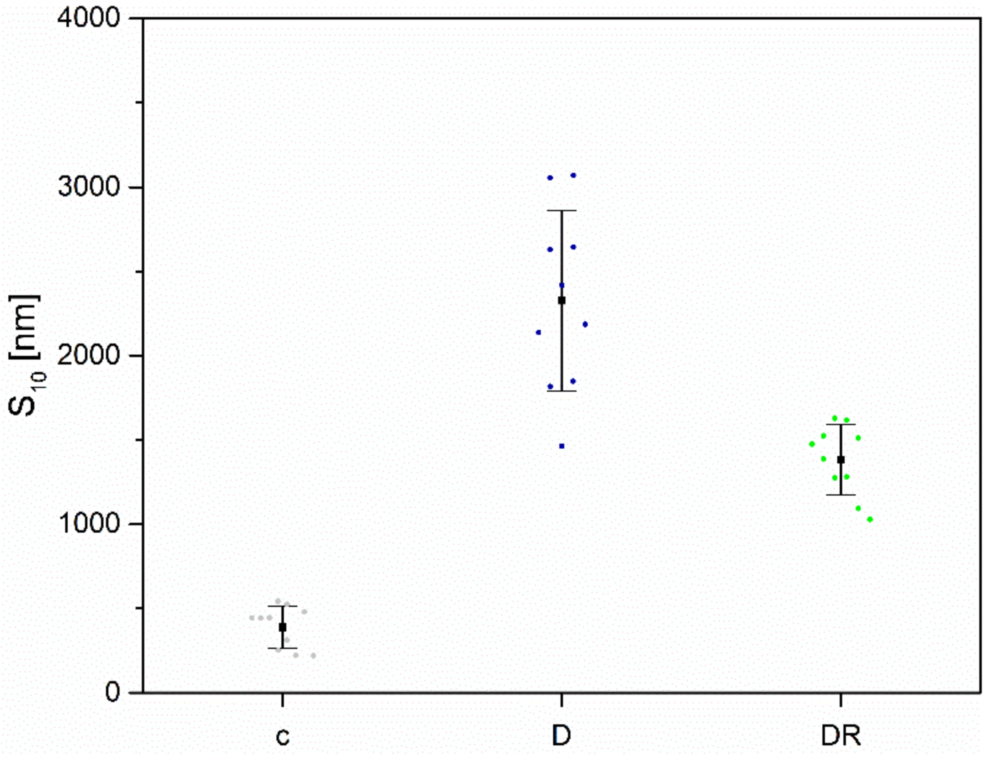

Figure S4: An averaged values of the average height calculated over five highest peaks and five deepest valleys (S_{10}) for the control (c) demineralised (D) and demineralised-remineralised (DR) enamel with corresponding standard deviations (whiskers) and mean values (black square).

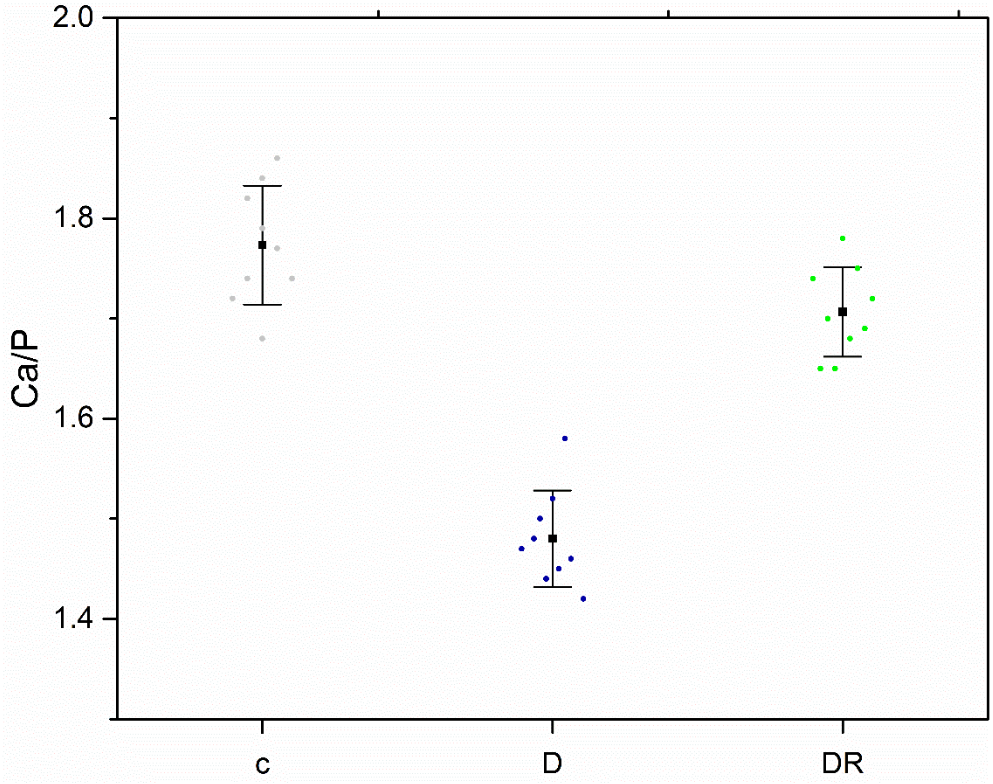

Figure S5: Ca/P ratios determined for the analysed samples from the control (c), demineralised (D) and demineralised-remineralised (DR) groups with corresponding standard deviations (whiskers) and mean values (black square).

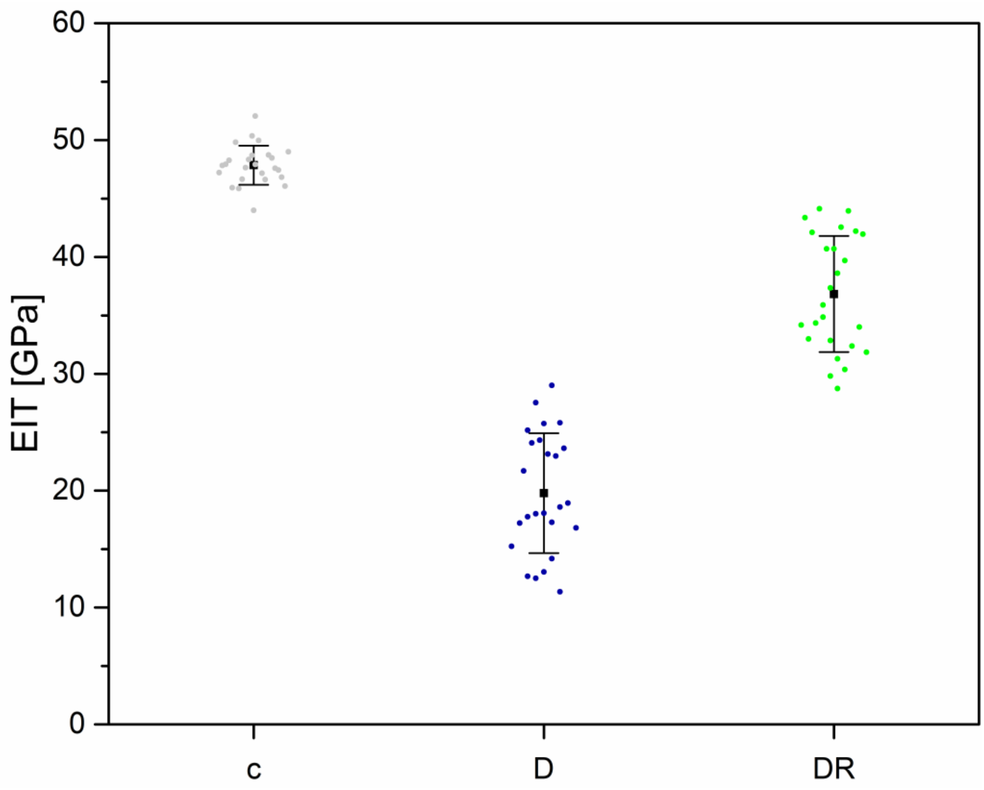

Figure S6: Indentation elasticity (EIT) values determined for the analysed samples from the control (c), demineralised (D) and demineralised-remineralised (DR) groups with corresponding standard deviations (whiskers) and mean values (black square).

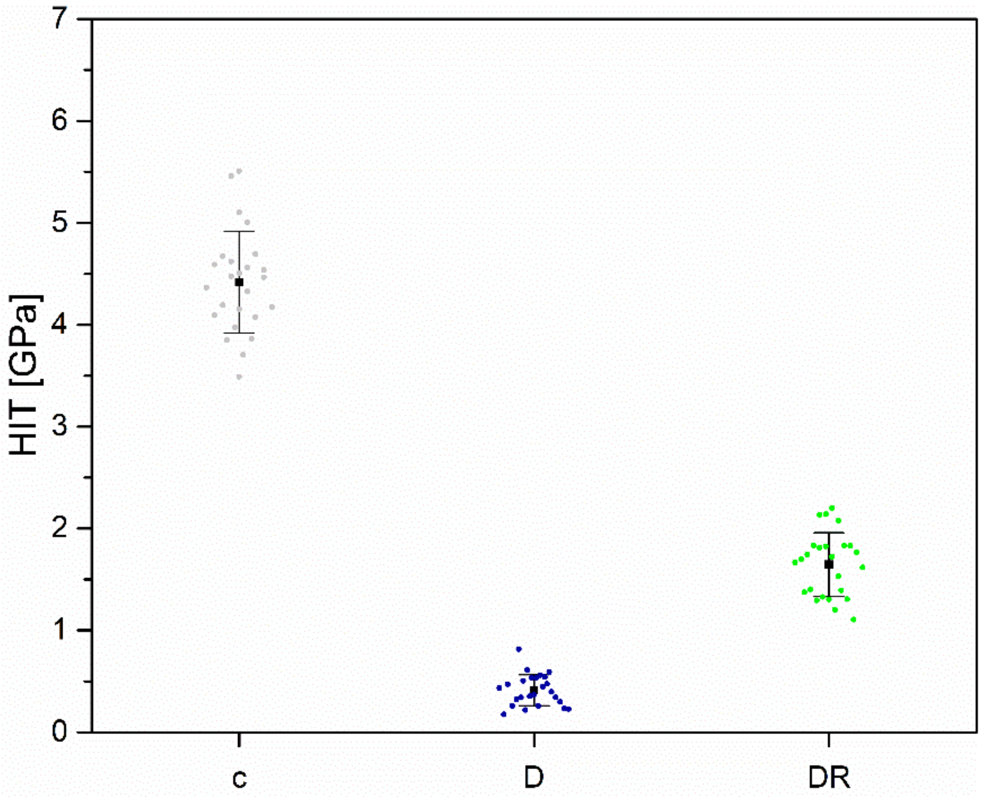

Figure S7: Indentation hardness (HIT) values determined for the analysed samples from the control (c), demineralised (D) and demineralised-remineralised (DR) groups with corresponding standard deviations (whiskers) and mean values (black square).
